# Supplementary material for: Identification of photoperiod-induced specific miRNAs in the adrenal glands of Sunite sheep (Ovis aries)
Source: Front Vet Sci. 2022 Jul 22;9:888207. doi: 10.3389/fvets.2022.888207 (PMC9354845; doi:10.3389/fvets.2022.888207)
Supplement: Supplementary file 1 [file Data_Sheet_1.ZIP › Supplementary file/miRDeep2 file.zip!/miRDeep2 file/Novel_Structure/SP42A2/Novel_637.pdf]

| | | |
|---|---|---|
| Provisional ID | : | NC_019484.2_43523 |
| Score total | : | 15.1 |
| Score for star read(s) | : | 3.9 |
| Score for read counts | : | 7.3 |
| Score for mfe | : | 2.4 |
| Score for randfold | : | 1.6 |
| Score for cons. seed | : | |
| Total read count | : | 26 |
| Mature read count | : | 25 |
| Loop read count | : | 0 |
| Star read count | : | 1 |

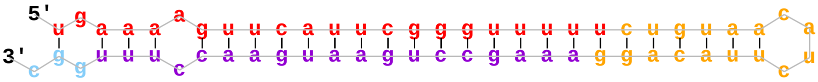


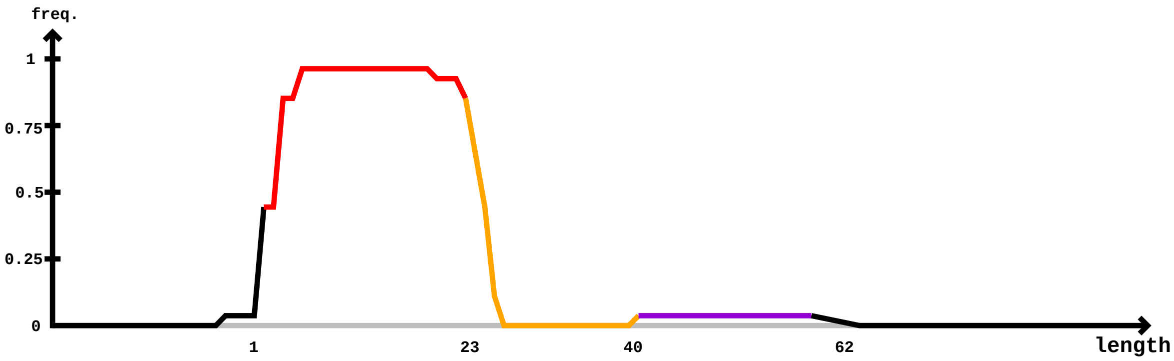


```
                     Mature                                        Star
5'- uccccacauauuggguuggcugaaaaguucauucggguuuuucuguaacaucuuacaggaaagccugaaugaaccuuuggccaacccaauauuuccgaccaguugcaagggc  -3'   obs
    uccccacauauuggguuggcugaaaaguucauucggguuuuucuguaacaucuuacaggaaagccugaaugaaccuuuggccaacccaauauuuccgaccaguugcaagggc        exp
    .......(((((((((((((((((..((((((((((((((((((((((....)))))))))))))))))))))).)))))))))))))))))......((........))..   reads   mm   sample
    ................uggcugaaaaguucauuUgggu..........................................................................   1       1    seq
    ....................ugaaaaguucauucggguuuu.......................................................................   2       0    seq
    ....................ugaaaaguucauucggguuuuu......................................................................   9       0    seq
    ......................aaaaguucauuUggguuuuuc.....................................................................   1       1    seq
    ......................aaaaguucGuucggguuuuucu....................................................................   7       1    seq
    ......................aaaaguucauuAggguuuuucug...................................................................   2       1    seq
    ......................aaaaguucGuucggguuuuucug...................................................................   1       1    seq
    ........................aaguucauucggguuuuuc.....................................................................   1       0    seq
    ........................aaguucauucggguuuuucu....................................................................   2       0    seq
    ...........................................................aaaAccugaaugaaccuuu..................................   1       1    seq
```
